# Etiological and predictive factors of pediatric urticaria in an emergency context

**DOI:** 10.1186/s12887-021-02553-y

**Published:** 2021-02-19

**Authors:** Leelawadee Techasatian, Pariwat Phungoen, Jitjira Chaiyarit, Rattapon Uppala

**Affiliations:** 1 Dermatology Division, Pediatric Department, Faculty of Medicine, Khon Kaen University, Khon Kaen, Thailand; 2 Emergency Department, Faculty of Medicine, Khon Kaen University, Khon Kaen, Thailand; 3 Department of Statistics, Faculty of Science, Khon Kaen University, Khon Kaen, Thailand; 4 Critical care and Pulmonology Division, Faculty of Medicine, Khon Kaen University, Khon Kaen, Thailand

**Keywords:** Urticaria, Pediatric, Children, Emergency, Etiology

## Abstract

**Background:**

Urticaria is common in pediatric population and is caused by various etiologies which usually differ among different age groups. The different etiologies require different management strategies. Thus, understanding detailed of the etiologies of urticaria in children would help pediatricians to perform appropriate initial treatment.

**Methods:**

A cross-sectional epidemiological study of all patients aged under 18-year-old with the diagnosis of urticaria from any causes entered in the emergency department during January 1st, 2016 to December 31st, 2019 by collecting the data from the Health Object Program®, an authorized electronic medical records program, at the Srinagarind Hospital, Faculty of Medicine, Khon Kaen University, Thailand.

**Results:**

There were total of 515 urticaria patients aged under 18 years old at the emergency department. The ages of patients ranged from 8 months to 18 years with a median age of 7 years (IQR 3.17–12.08). The majority of the patients were in the preschool-aged group (40.97%), followed by the school-aged (28.16%), adolescent (22.14%), and infant (8.74%). Six major etiologic categories were identified in the present study. The most common cause of urticaria was infection (51.26%), followed by idiopathic urticaria (34.37%), inhalants (6.99%), drugs (4.08%), foods (2.52%), and insect stings (0.78%).

**Conclusions:**

Having underlying allergic diseases had a strong association with all identified causes of urticaria in the study population, of which, food and inhalation etiologies had a significant difference when compared to the other identified causes. The present study has found that infection was the most common cause of acute urticaria in children. This etiology (infection-induced urticaria) usually presents concurrent with fever, however, non-febrile symptoms were also presented. Therefore, in the pediatric population, pediatricians should always look for infection as the cause of urticaria even in patients without pyrexia.

## Introduction

Urticaria is common in pediatric population and is one of the most common dermatologic conditions presenting at the emergency departments [1]. The prevalence of urticaria in children ranged from 15.3 to 22.5% [2]. Even though the majority of patients with urticaria are usually benign, however, this problem can cause parental concern especially with the first episodic attack. Urticaria and angioedema can be an early sign of anaphylaxis which need an immediate treatment [3–5]. The diagnosis of urticaria can be made by its typical presentation of acute edematous, wheal and flare appearance, sometimes also with angioedema. It has its fleeting nature (may be absent by the time patients are actually seen). The lesions are usually itchy. The diagnosis can be challenging in some circumstances conditions which can mimic urticaria (e.g. viral exanthem, morbilliform drug eruption, insect bites, contact dermatitis, etc.) [6]. The acute episodic form is the most prevalent in the pediatric population, and it is often a recurrent phenomenon [7]. Urticaria and angioedema are caused by various etiologies which usually differ among different age groups [8–10]. These included; infections (virus, bacteria, fungus, and parasite) [11–13], allergic reactions to foods and drugs, or physical stimuli. In many instances it is not possible to identify a specific cause (idiopathic urticaria). The different etiologies require different management strategies. A recent publication has found etiologies related to medications or infections, a history of allergies, and the co-occurrence of pyrexia or urticaria were predictors of angioedema [14]. Thus, understanding the details of the etiologies of urticaria in children would help pediatricians to perform appropriate further treatment. This led to the objective of the present study to explore the etiology of urticaria in children in the emergency department and find out the predictive factors associated with different etiologies of pediatric urticaria that may help pediatricians in managing urticaria in an emergency setting.

## Method

### Study design

The authors conducted a cross-sectional epidemiological study from January 1st, 2016 to December 31st, 2019 by collecting the data from the Health Object Program®, an authorized electronic medical records program, at the Srinagarind Hospital, Faculty of Medicine, Khon Kaen University, Thailand. All patients aged under 18-year-old with the diagnosis of first episode urticaria from various causes, entered at the emergency department, Faculty of Medicine, Khon Kaen University were included in to the study.

The study was approved by the institutional review board of the Khon Kaen University, Human Research Ethics Committee (#HE631510). The waiver for the informed consent was approved and all methods were performed in accordance with the relevant guidelines and regulations.

### Statistical analysis

At the end of the study, the collected data were analyzed using STATA software version 10 (StataCorp LP). Descriptive statistical methods, means, standard deviations (SDs), medians and frequencies were used to analyze the demographic data. Values of *P* < .05 were considered to indicate statistical significance. Multinomial logistic regression and post hoc analysis were used to test for the association between etiologic factors of urticaria.

## Results

There were total of 515 urticaria patients aged under 18 years old at the emergency department during the study period. There were 283 (54.95%) male and 232 (45.05%) female patients; thus, the ratio of males to females was 1.2. The ages of patients ranged from 8 months to 18 years with a median age of 7 years (IQR 3.17–12.08). The age range were classified in to four groups; infant (< 1-year-old), preschool-aged (1 to 6-year-old), school-aged (7 to 12-year-old), and adolescent (13 to 18-year-old). The majority of the patients were in the preschool-aged group (40.97%), followed by the school-aged (28.16%), adolescent (22.14%), and infant (8.74%). There were 68 patients (13.2%) with fever and 27 patients (5.24%) with angioedema. The patients’ underlying allergic diseases (atopic dermatitis, allergic rhinitis, and asthma) were documented in 59 patients (11.45%). Majority of patients (79.42%) had their onset of urticaria less than 24 h prior to visiting the emergency room. Table 1 shows demographic, clinical presentations, and onset of urticaria in the study population.

**Table 1:** Demographic data of acute urticaria in children from January 1st, 2016 to December 31st, 2019

Variable	Total (*n* = 515)	Year			
		2016 (*n* = 99)	2017 (*n* = 149)	2018 (*n* = 149)	2019 (*n* = 118)
Sex					
Male	283 (54.95)	57 (57.58)	74 (49.66)	80 (53.69)	72 (61.02)
Female	232 (45.05)	42 (42.42)	75 (50.34)	69 (46.31)	46 (38.98)
Age (years)					
Median (IQR)	7.08 (3.17–12.08)	6.42 (3.17–11.67)	7.25 (3.33–12)	6.75 (2.92–12.25)	7.79 (2.75–12.58)
Median (Min-max)	7.08 (0.08–17.92)	6.42 (0.67–17.92)	7.25 (0.33–17.92)	6.75 (0.08–17.83)	7.79 (0.25–17.67)
Mean ± SD	7.83 ± 5.43	7.4 ± 5.36	8 ± 5.53	7.66 ± 5.29	8.18 ± 5.58
Age					
< =1 yr	45 (8.74)	5 (5.05)	14 (9.4)	10 (6.71)	16 (13.56)
1-6 yr	211 (40.97)	50 (50.51)	60 (40.27)	66 (44.3)	35 (29.66)
7-12 yr	145 (28.16)	24 (24.24)	41 (27.52)	39 (26.17)	41 (34.75)
13-18 yr	114 (22.14)	20 (20.2)	34 (22.82)	34 (22.82)	26 (22.03)
Fever					
No	447 (86.8)	86 (86.87)	129 (86.58)	133 (89.26)	99 (83.9)
Yes	68 (13.2)	13 (13.13)	20 (13.42)	16 (10.74)	19 (16.1)
Angioedema					
No	488 (94.76)	94 (94.95)	147 (98.66)	140 (93.96)	107 (90.68)
Yes	27 (5.24)	5 (5.05)	2 (1.34)	9 (6.04)	11 (9.32)
U/D Allergy					
No	456 (88.54)	88 (88.89)	135 (90.6)	131 (87.92)	102 (86.44)
AD	34 (6.6)	8 (8.08)	8 (5.37)	12 (8.05)	6 (5.08)
AR	25 (4.85)	2 (2.02)	4 (2.68)	7 (4.70)	12 (10.17)
Asthma	5 (0.97)	1 (1.01)	2 (1.34)	1 (0.67)	1 (0.85)
Discharge type					
Discharge	508 (98.64)	97 (97.98)	148 (99.33)	148 (99.33)	115 (97.46)
Admission	7 (1.36)	2 (2.02)	1 (0.67)	1 (0.67)	3 (2.54)

**Fig. 1:**
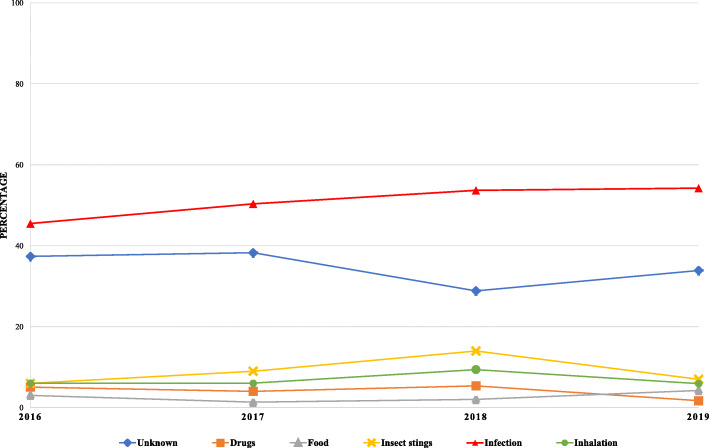
Prevalence of various etiologies of acute urticaria in children from January 1st, 2016 to December 31st, 2019

Six major etiologic categories were identified in the present study. The most common cause of urticaria was infection (51.26%), followed by idiopathic cause (34.37%), inhalants (6.99%), drugs (4.08%), foods (2.52%), and insect stings (0.78%). We did not find acute urticaria caused by physical stimuli from the present study. Figure 1 shows prevalence of various etiologies of acute urticaria in children from January 1st, 2016 to December 31st, 2019.

**Fig. 2:**
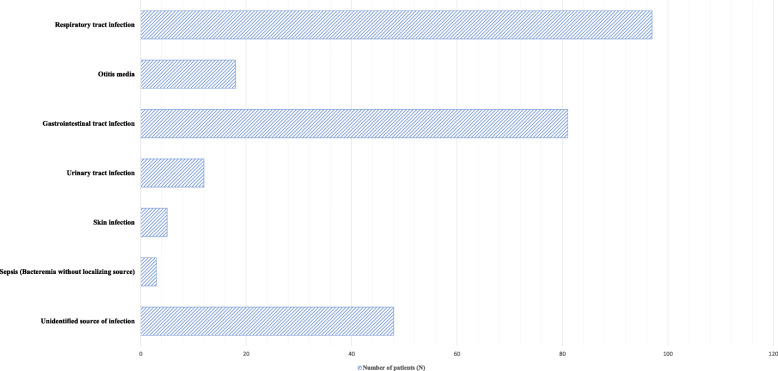
Number of patients with various sources of infection in infection-induced urticaria in the study population

There were various sources of infection in infection-induced urticaria in the study population. Respiratory tract infection was the most common cause (97 cases, 36.74%), followed by gastrointestinal infection (84 cases, 31.82%), unidentified source of infection (48 cases, 18.18%), and otitis media (18 cases, 6.82%). Figure 2 showed number of patients with various sources of infection in infection-induced urticaria in the study population.

There were 59 patients (11.45%) with at least one underlying allergic disease (atopic dermatitis, allergic rhinitis, and asthma). Multinomial logistic regression and post hoc analysis revealed an association between urticaria (caused from foods, inhalation, drug, insect stings, and infection) and patients with underlying allergic diseases (*P* < 0.001). Two etiologies of urticaria (foods and inhalation) showed a significant difference from other causes (drug, insect stings, and infection), Table 2.

**Table 2:** Association test among factors and the cause of urticaria by using multinomial logistic regression analysis

Factor	Cause of urticaria					*p*-value
	Drugs (*n* = 21)	Foods (*n* = 13)	Insect stings (*n* = 4)	Infection (*n* = 264)	Inhalation (*n* = 36)	
**Angioedema**						0.659
No	19 (90.48)	13 (100)	4 (100)	251 (95.08)	33 (91.67)	
Yes	2 (9.52)	0 (0)	0 (0)	13 (4.92)	3 (8.33)	
**Underlying allergic diseases**						< 0.001
No	19 (90.48)	1 (7.69)	4 (100)	252 (95.45)	4 (11.11)	
Yes	2 (9.52)^a^	12 (92.31)^b^	0 (0)^a^	12 (4.55)^a^	32 (88.89)^b^	
**Onset of chief complaint**						0.438
≤ 24 h	20 (95.24)	10 (76.92)	3 (75)	209 (79.17)	27 (75)	
> 24 h	1 (4.76)	3 (23.08)	1 (25)	55 (20.83)	9 (25)	

Different superscripts (a, b) in the same row indicate a significant difference between groups (chi-squared test, multinomial logistic regression analysis, *p* < 0.05)

## Discussion

Urticaria is common in pediatric population. The onset of rashes are usually acute in nature, thus, majority of the first attack acute urticaria patients usually visit emergency department [10, 15, 16]. The cutaneous rash has typical presentation of wheal edematous of the dermis, mostly itch, and some with angioedema [1]. In newborns and infants, acute urticaria is less common, is typically generalized, and features large annular or geographic plaques which are often slightly raised [17]. Some propose that differences in the prevalence and morphology in this age group are due to functional insufficiency of the immune system and skin characteristics [17].

Urticaria is more common in adults and is usually idiopathic or spontaneous [18, 19]. In contrast, acute urticaria in children is mostly caused by infection [2]. A recent report of the on going COVID-19 pandemic had found that acute urticaria and pyrexia can be the first manifestations of a COVID-19 infection [20]. Previous reports have found that urticaria and angioedema are caused by various etiologies which usually differ among different age groups [2, 7, 9]. This finding is similar to the present study that revealed a significant difference in the prevalence of various urticaria etiologies between different age groups (Table 3). Even though there were various causes, however, the most common cause of urticaria in the overall pediatric population was infection. This is correlated to the results from previous studies that have found infection, especially viral infection, as the most frequent cause in pediatric population [2, 7, 21]. Infection-induced urticaria is thought to involve the complement system and plasma effector systems [10], while the others; foods, drugs, inhalation cause urticaria via an IgE- and IgE receptor-dependent mechanism. Infection-induced urticaria is more common in young children. This finding is perceived in a similar way to the present study that in infants and preschool-aged group, infection was the most common cause, while in the older age groups (school-aged and adolescent), unknown cause (idiopathic urticaria), drugs, and inhalation were found be more frequent. Thus, history taking of exposures (drugs, foods, inhalation) to identify causes of urticaria in older children should not be missed.

**Table 3:** Number (%) of patients classified by the cause of urticaria in four different age-groups

Age	Total (*n* = 515)	Cause of urticaria					
		Idiopathic (*n* = 177)	Drugs (*n* = 21)	Foods (*n* = 13)	Insect stings (*n* = 4)	Infection (*n* = 264)	Inhalation (*n* = 36)
Median age (IQR), years	7.08 (3.17–12.08)	7.08 (3.17–12.08)	12.58 (9.42–15.83)	7 (4.58–15.83)	1.25 (0.83–1.92)	16.83 (9.125–17.455)	3.96 (1.58–6.75)
<=1 yr	45 (8.74)	1 (0.56)	0 (0)	6 (46.15)	0 (0)	37 (14.02)	1 (2.78)
1-6 yr	211 (40.97)	15 (8.47)	10 (47.62)	5 (38.46)	1 (25)	165 (62.5)	15 (41.67)
7-12 yr	145 (28.16)	78 (44.07)	3 (14.29)	2 (15.38)	0 (0)	51 (19.32)	11 (30.56)
13-18 yr	114 (22.14)	83 (46.89)	8 (38.1)	0 (0)	3 (75)	11 (4.17)	9 (25)

Chi square test (15) = 247.0309, p-value < 0.001

Sixty-eight patients (13.2%) had fever, of which, infection-induced urticaria was diagnosed in 62 patients. However, infection-induced urticaria can be found in nonfebrile patients. This may due to the fact that, patients can develop urticaria at a time of infection or days to weeks after. Thus, normal temperature in infection-induced urticaria can be found. Therefore, in the pediatric population, pediatricians should always look for infection as the cause of urticaria even in patients without pyrexia. The present study performed a correlation test between fever and the cause of urticaria by using multinomial logistic regression analysis, nevertheless, no associations were found.

Angioedema was found in 27 patients (5.24%). This symptom can indicate an early sign of anaphylaxis. Angioedema can be found together with urticarial rashes or else as an isolated finding [4]. The present study revealed that this finding is not associated to any causes of urticaria, Table 2. However, angioedema can be an initial sign of anaphylaxis that need a prompt treatment [22–26]. Therefore, if a clinical sign of angioedema is documented, physician should prepare for the possible severe presentation.

Onset of the rashes in urticaria patients usually present as acute onset in nature. The authors tried to find the association between the onset of rashes prior to the emergency room of less and more than 24 h and the etiology of urticaria. However, this factor has no correlation to any urticaria causes, Table 2.

Underlying allergic diseases (atopic dermatitis, allergic rhinitis, and asthma) were documented in 59 patients (11.46%). A recent publication among a large cohort study in adolescent has found a strong association between chronic spontaneous urticaria and atopic diseases [27]. These association were similar to the present study that also found an association between identified cause of urticaria (food, inhalation, drug, insect stings, and infection) and underlying allergic diseases, *P* < 0.001 (Table 2). Among the five different groups of etiologies, urticaria caused by food and inhalation, showed a significant association with allergic disease when compared to the other causes (drug, insect stings, and infection) in the study population.

From January 2016 to December 2019, etiologies of urticaria in children did not change over time (Fig. 1). Infection was always the leading etiology in pediatric population, thus investigation of underlying infections is essential in this age group.

### Limitation

The main limitation of the present study was the retrospective study design. Some of the data recorded was in a structural form, as it was an electronic file, which resulted in a lack of some information. For example, the timing of onset and persistence of urticaria could not be determined when physicians did not add this information in the examination. Another limitation of this study is that it relied on physicians in the emergency room to establish the diagnosis of urticaria and its cause. Without additional details, the accuracy of these assessments could not be confirmed. In many cases where urticaria was attributed to infection, it was unclear whether other febrile conditions with rash, viral exanthem or drug-induced urticaria (e.g. NSAID-induced) were fully excluded. Moreover, some essential information in some patients, such as history of allergy was also missing. In patients who had inhalants allergens (e.g. pollens, grasses, dust mites) listed as the cause of their urticaria, it was not clear whether this occurred through contact or other routes.

## Conclusions

The present study revealed several identified causes of urticaria (food, inhalation, drug, insect stings, and infection). All etiologies had a strong association with the underlying allergic diseases, of which food and inhalation causes had a significant difference when compared to the other identified etiologies. The present study has found that infection was the most common cause of acute urticaria in children. This etiology (infection-induced urticaria) usually presents concurrent with fever, however, probable non-febrile symptoms can also exist. Therefore, in pediatric population, pediatricians should always look for the underlying causes of urticaria from infections even in patients without pyrexia symptoms.

## Data Availability

The datasets generated and/or analysed during the current study are not publicly available, but available from the corresponding author [LT and RU] on request.
